# Clinical Notes from La Charité, of Paris

**Published:** 1879-03

**Authors:** W. S. Caldwell

**Affiliations:** Berlin


### Foreign Correspondence.

### ARTICLE XIII.

### CLINICAL NOTES FROM LA CHARITÉ, OF PARIS.

The two most popular teachers of clinical medicine in Paris are Prof. Germain Sée, of the Hôtel-Dieu, and Prof. Hardy, of La Charité. The wards and lecture rooms of both these men are always crowded. The former is perhaps more widely known as a writer and lecturer than the latter, but among the students and members of the profession generally in Paris, Prof. Hardy is looked upon as the more reliable teacher of the two. While Prof. Sée is ever launching out into some new field in pathology and therapeutics, Prof. Hardy is more inclined to teach thoroughly such facts in medicine as are well settled and beyond controversy.

Enquiring of a French professor in another department of medicine as to which of these men would prove most beneficial as an instructor, he said, "Prof. Sée is a man who has always a hobby, and consequently his teachings are not always reliable. On the other hand, Prof. Hardy is ever trustworthy, and his observations can be more implicitly relied on." As the readers of the JOURNAL will probably remember, in 1877 I gave Prof. Sée as authority for some glowing statistical results of the treatment of rheumatism with the salicylate of soda. Since that time I have taken some pains to compare these with those obtained in the other hospitals of Europe that I have visited, and while such results are not generally so good as those given by him, I think it can be safely said that we have in this remedy an agent far superior to anything before known to the profession as an anti-rheumatic.

Among the new ideas that Prof. Sée enthusiastically enunciated during the first part of his lecture course last fall, was the curability of certain forms of cirrhosis of the liver by the administration of large doses of the iodide of potassium, and the successful treatment of morbus Basedowi by the continued use of the tincture of veratrum viride.

Prof. Hardy, in his introductory address to his class, laid great stress upon the importance to every student of medicine of his being well grounded in two subjects: *pathology* and *diagnosis*. These two branches of medical science, he said, were the compass and the rudder that would guide to the desired haven in our profession. Without them, one will find himself adrift upon an unknown sea, beat about by every changing wind. While the way to study pathology was clear and apparent to all, and men would generally pursue it with tolerable diligence, he found considerable difficulty often in making good diagnosticians of his students.

The first cause of this failure was that many could not be made to comprehend the importance of the subject. But the second and most general reason for failure was that they early imbibed a notion that they were possessed of a certain intuitive instinct that enabled them to diagnosticate a case after the most superficial and cursory examination. "I have seen," he says, "all through life, men old and young who claimed to be endowed with such an intuition; but I can assure you that it was always a mistaken idea. Every now and then these men make some woeful blunder, that blasts their own fame and brings disgrace upon our profession. That man will ever make the best diagnostician who studies his subject most thoroughly and thinkingly, and observes with the most scrutinizing eye every morbid phenomenon in the case that he is called to investigate."

### SCARLATINA.

An adult female patient, aged 37 years, was admitted to La Charité, Nov. 20th, 1878, suffering from a tolerably severe attack of scarlet fever.

Prof. Hardy, in lecturing at the bedside of the patient, made in substance the following remarks worthy of note:

"Of the exanthemata, this disease is least likely to recur a second time. Treatment should be expectant or symptomatic. In all mild cases we have absolutely *nothing* to do. It is only when we have the supervention of some complication that we are called on to interfere. Among the complications that may arise in the course of every case, and especially every severe one, and which will call for our active interference, is that of an extremely high temperature. During my professional life I have watched many cases anxiously, and all went well until suddenly a temperature of 40.5 or 41 was attained.

"If I happened to see the patient only a short time after the supervention of such hyper-pyrexia, there would be perhaps nothing else about the case to excite apprehension or alarm. I would leave the house in the evening with a promise to call the next morning, but within the next few hours, say from six to ten after my departure, I would be summoned again, to find my patient either wild and delirious or in a condition approaching that of coma.

"After this state of things supervened, I had not seen for years a single case recover, until I adopted the course of treatment which I shall presently recommend. It was formerly thought that these nervous manifestations, and especially that of stupor, were caused by uræmic poisoning.

"But later investigations have proven that this is not the case. They have demonstrated that all kidney complications belong to the later or desquamative stage of the disease.

"These symptoms, denoting a fatal implication of the nervous centers, are produced by extreme heat of the body, and to the combating of this condition we must direct our treatment.

"What are we to do? Give medicines? I can assure you that we have no remedy in the materia medica that will exert the least influence in preventing a fatal termination in such a case as this. There is one thing, and one only, that you can do with a hope of saving life, and that is, immerse the patient at once in a cold bath of from 18° to 20°, and repeat this bath sufficiently often to bring the temperature down. If you do this early, and do it thoroughly, you may be successful. If you vacillate, your patient will assuredly die.

"Great care should be taken in the after management of scarlatina in cases of adults, for while they generally have the disease in a much milder form than do children, and are less likely to die in the acute stage of the disease, they are much more likely to have nephritis as a sequel, and such a complication with them is much less amenable to treatment than among patients during the first years of life.

"My rule is to confine children to the house for one month after an attack of scarlet fever, and if adults follow my advice, they will not expose themselves to any vicissitudes of temperature for at least two months after apparent recovery from this disease."

### TYPHOID FEVER.

On November 21st, 1878, Prof. Hardy lectured on the following case:

Jean Drowin, aged 17 years, was admitted to the wards of La Charité Nov. 14th. When questioned as to his symptoms, he simply complained of a general feeling of malaise; he suffered no pain, and nothing of a morbid character could be discovered by the most careful physical examination. He had no diarrhœa nor tympanites, but had some appetite.

"When this patient came under observation, we had to hold our diagnosis in reserve. In fact, we were unable to tell with what disease we had to deal. His temperature for the first five days following his admittance I have had written out on that large card placed upon the wall, so that all may see it at a glance."

It was as follows:

| | | | | |
|---|---|---|---|---|
| Nov. 14th, | morning, | 37.4; | evening, | 38.2. |
| " 15th, | " | 37.6 | " | 38.7. |
| " 16th, | " | 38. | " | 39. |
| " 17th, | " | 38.4 | " | 39.9. |
| " 18th, | " | 38.7 | " | 40. |
| " 19th, | " | 38.8 | " | 40.1. |

"This temperature curve, gentlemen, corresponds to but one disease, and that is typhoid fever. To further verify our diagnosis, I will heat a small portion of the patient's urine, which you see contains a trace of albumen.

"Another point that would assist us if we were still in doubt as to the nature of the disease with which we have to deal, is the fact that this lad had been only six months in Paris, and according to my observation, persons of this age who come to this city from the provinces and inhabit the quarter in which he lives, are very likely to have an attack of typhoid fever as a kind of acclimating process.

TREATMENT.

"If the last quarter of a century has taught us anything in medicine, it is this fact, that all active treatment in the management of infectious diseases, such as the one under consideration, erysipelas and the like, with a view of cutting them short, is utterly futile, and if such treatment disturbs in any way the natural functions of the body, it is absolutely injurious.

"When this patient entered the hospital he was costive, and hence we gave him a mild cathartic of carbonate of magnesia and syrup of rhubarb. More than this, we did not think it best to do at the present time. We find that his temperature has a tendency to remain at and exceed 40° during the exacerbation of his fever. We shall now direct our attention to the reduction of this patient's temperature.

"To effect this object we shall use cold water. The next question that arises is how are we to use this agent. Many, especially the Germans, are in the habit of immersing their patients in a cold bath. To this practice I object on two accounts:

"First, to carry it out well, we must have bath-rooms and bath-tubs annexed to each ward, thus increasing the expense and labor connected therewith.

"Second and chiefly, I object to repeated baths for typhoid fever patients on account of the disturbance and muscular effort that is connected with their administration.

"Instead of this practice we shall have our patient sponged off with cold water of from 10° to 15° every two to four hours during the height of his fever. But a practice that I have lately followed, and which I can strongly recommend is the injection of cold water into the rectum. For this purpose I use water of about 12° and to each injection I add two grams of salicylic acid. The quantity that I use is from 500 to 1,000 grams, according as the patient can contain it. These injections we try to induce the patient to retain from thirty to sixty minutes and repeat them every four to six hours. Some have even recommended them to be given at the freezing point, but this extreme cold we have always found to disturb the patient's nervous system."

Prof. Bernutz who has charge of the gynæcological wards in this hospital, has also one male ward that is devoted largely to typhoid fever cases. I followed him for three months and will give the two following cases as examples of his management of this disease:

Y. L., aged twenty-seven years, a waiter in a café, was admitted to the wards Sept. 15th, giving a history of diarrhœa and alternate sensations of heat and chilliness for ten days before he entered. On the day of his entry his morning temperature was 38 and evening 39.1. For the next six days after his admission the course of the disease was not marked by any special phenomenon, except that his diarrhœa was rather excessive, the discharges amounting to from eight to twelve in the twenty-four hours. The entire treatment during this time consisted of one half gram each of sulphate of quinia and tannin given night and morning.

On the 25th, or ten days after he came in, the slight bronchial irritation from which he had lately suffered became worse, he was harrassed by an irritating cough so that he got but little sleep day or night. At this date, a solution of the acetate of ammonia, sweetened to a syrup and to which was added a small quantity of ipecacuanha, was prescribed as an expectorant. On the 27th the lower lobe of the right lung was found consolidated, his pulse was 130 and weak, his temperature was 40.2 and his hands were slightly cyanotic. On the 28th his pulse was 135, temperature, 40.5, respiration 40, and the cyanotic condition of his extremities was more marked.

He was now ordered sixty grams of brandy and two grams of quinia every twenty-four hours. The patient gradually grew worse and died on the 30th.

On the 1st of October the autopsy showed both lower lobes of the right lung in a condition of red hepatization, the spleen enlarged to four times its natural size, studded with infarcted spots and soft in texture. The glands of Peyer in the vicinity of the cæcum, extending also for one and a half centimeters into the ileum, were either ulcerated or congested and swollen.

On October 15th, G. Golosko, a Russian, aged 21, was brought from his hotel into one of the private wards of La Charité, under the service of Prof. Bernutz. He had been sick for two weeks before he was admitted, and had the petechial eruption and other distinctive symptoms belonging to a true case of typhoid fever. He had but little diarrhœa, but suffered extremely from headache and an harrassing, tickling cough. His morning temperature was 38.2, evening 39.6. He was put on a slightly acidulated solution of muriatic acid, flavored with lemon-juice, and one gram of quinia in the twenty-four hours. The acid given was not in a sufficiently large dose to be considered in the light of any medication. The patient not being able to speak French, but speaking German, I acted as interpreter between him and his physician and nurse, and so had to visit him two or three times a day.

On the second day that I made my visit to him in company with Prof. B., he appealed to me for the sake of heaven to ask the doctor to give him something to ease his head and check his cough. Communicating his wishes to the Professor, he replied that " all was being done for him that could be done." I then asked (in a modest way) whether some mild form of opiate, say a Dover's powder at night, might not act well by easing the patient's headache and arresting his cough. His reply was : " I never give opiates or any form of anodyne in typhoid fever on account of its tendency to obstruct the action of the heart. I think it a matter of the first importance that nothing should be done in this disease to cripple the great center of circulation, and this I think opiates have a tendency to do."

Some years ago a celebrated Professor of materia medica, by means of the sphygmograph, demonstrated to me that the effect of both opium and digitalis upon the circulatory system, is to increase the muscular contractility and force of the heart's action, as well as the blood pressure in the arteries ; yet this venerable old man, who has practiced his profession for half a century, and has made a name that is known the wide world over, tells me positively that both these remedies have exactly the opposite effect to that which I have been taught to believe that they possessed.

This patient began to convalesce slowly on the 30th of October, and was discharged on the 16th of November, or one month after his admittance.

### ACUTE RHEUMATISM.

Léon Patry, aged 21 years, a painter by trade, was admitted to LaCharité Oct. 10th, 1878, suffering from acute poly-articular rheumatism. Eight days before his admission, he began to have pains in his joints, chilly sensations when he came in contact with currents of cold air, and a general feeling of malaise. On making his regular visit to the wards, at the date above mentioned, Dr. Dieulafoy (inventor of the aspirator that bears his name) lectured at the bed side of this patient. He said:

"This is a good type of acute articular rheumatism. The patient had some ill-defined symptoms for several days previous to his entry into the ward, but three days ago he was taken violently ill. His knee and ankle joints on both sides are much swollen and extremely painful. His temperature is 40.3, his pulse is 130, and his respirations are 45 per minute. From the anxious aspect of this patient and his extreme dyspnœa, it is evident that he is suffering from some impediment to his respiration, his circulation, or both. The careful physical examination of his case which I have just made, and which, if the patient is not too much fatigued, I will allow you all to verify, reveals the following conditions: Placing the stethoscope at the junction of the fourth rib, with the sternum on the left side, we hear a dry friction sound *bruit de frottement*. This sound is synchronous with both the systole and diastole of the heart. By making a pretty firm pressure with the stethoscope it is considerably increased in intensity. This sound to which I first call your attention, is peri-cardial in character, and, being a dry sound, it proves the existence of pericarditis in the first stage. I want you all to study this sound well, not only now, while we have the first stage of this disease, but later when the sound will probably be altered in character by an effusion of fluid into the peri-cardial sac. It is one of the conditions that men who are not skillful in physical diagnosis constantly overlook. If we place the stethoscope over the point where we see the apex beat, we hear another sound, *bruit de souffle*, which is synchronous with the ventricular systole. The sound that we hear at this point is endo-cardial in character, and is produced by an impairment of the function of the mitral valve, showing the existence of an endo-carditis. We will now allow the patient to sit upright, and examine his chest to see whether we cannot find some lesion in this region. Synchronous with the respiratory movements of the lungs, we hear a friction sound on both sides, which is dry throughout on the left side, but is moist at the lower portion of the right.

"We have then in this case, beside the general affection of the joints, a peri-carditis, an endo-carditis, and a double pleurisy with a slight effusion into the right pleural cavity. A point to which I wish to draw your attention in connection with this case is the morbid phenomena that we have found situated in thoracic cavity. By most writers and teachers, whenever a rheumatic inflammation attacks any part of the body other than the articular surfaces of the joints, we are taught that such manifestations of the disease are to be looked upon as accidents or complications. This idea is an entirely erroneous one. The disease may attack any of the fibrous tissues of the body, but has a preference for the fibrous structures that go to make up the linings of the various serous cavities. Therefore an endo-carditis is no more a complication of rheumatism than an inflammation of any one of the joints of the extremities.

"The pleurisy we have in this case is one of the accompaniments of this disease that may be very easily overlooked, for, strange as it may seem, the suffering from this form of pleuritis is not as great as in other forms of acute pleuritic inflammation. The effusion is also much more likely to be rapidly absorbed than where it originates from other causes.

### TREATMENT.

"The first remedy, that occurs to every physician at the present day, in connection with the management of a case of acute rheumatism, is the salicylate of soda. In this case we will not give this drug, and for this reason. Its physiological action is to lessen the contractile force of the heart and to decrease the blood pressure in the capillaries; and as we have in this case already a crippled heart, accompanied by a cyanotic condition of the skin in the extremities, we consider the salicylates as contra-indicated. Were the heart and its investing membranes unaffected, we should give this remedy in full doses, and expect a good result from its administration. We shall give this patient for the next twenty-four hours:

| | | |
|---|---|---|
| Digitalis leaves | | 50 |
| Sodic bicarbonate | 4 | |
| Quinia sulphate | 2 | |
| Camphor | | 20 |

"The digitalis to be infused in 100 grams of water, one-fourth to be taken every 6 hours. The bicarbonate of soda we will give also in four dosés, each dissolved in 200 grams of water, flavored with syrup of raspberry. The quinia we will give in 0.50 gram doses, and the camphor we shall give in two doses, one night and morning."

The record of this patient's highest temperature, respiration and pulse was as follows for the days here given:

Oct. 11th.—Temperature, 40; pulse, 125; respiration, 46.

Oct. 12th.—Temperature, 39.5; pulse, 121; respiration, 44.

Oct. 15th.—Temperature, 38.6; pulse, 104; respiration, 34.

At the last date the quinia was reduced to one gram in the 24 hours.

Oct. 22d.—Temperature, 38; pulse 100; respiration, 25. At this last date the acute symptoms had mostly subsided. The patient was free from pain, there was a slight peri-cardial and pleuritic effusion, and the endo-cardial murmur was a little more accentuated than when at the first examination. The sodic bicarbonate was now discontinued, and the iodide of potassium given in its stead. He was also given a small amount of claret wine.

Oct. 31st.—Temperature, 37.2; pulse, 90; respiration, 20. The patient was now pretty comfortable sitting up in bed, but had still a loud mitral, regurgitant murmur. I omitted to mention in the right place that the patient had a large cantharides blister applied over the heart, on the 12th of October. He left the hospital on Nov, 9th, free from pain but feeble and still showing the mitral lesion that came on during the first days of the attack.

I will close this article with the following brief account of a case of acute rheumatism that I have under observation at this date, Feb. 1st., at the Charité, of Berlin. J. Kapp, a young man aged 20 years, was admitted on the 9th of January last. He had always enjoyed good health excepting a former attack of this disease, for which he had been treated in this institution two years ago, and from which he entirely recovered.

Prof. Frerichs brought this patient into the lecture-room on Jan. 11th, and the following is a brief summary of some of the points made in his discourse at his bedside.

The disease is not generally ushered in by a chill. It usually approaches somewhat insidiously, so that the patient is unable to say exactly when he was taken ill. The nature of the disease has been a subject of controversy from the earliest times.

Chemical changes in the blood, small emboli going out from an ever supposed endo-carditis, and an infectious poison are among the causes that are supposed by different authors to produce this affection.

As to the first two supposed causes, they rest on hypotheses only, with no proof whatever to substantiate their truth. As to its being an infectious disease, that he thought entirely improbable, as the affection had no regular course or limit of duration, which was a characteristic of diseases of that type.

The best course, in the present state of our knowledge on this subject, is to own frankly that we do not know what is the true and intrinsic cause of this disease.

### TREATMENT.

There is no disease in which it is so difficult to test the efficacy of remedies, as the one we have under consideration. The reason of this is obvious. The case may terminate in spontaneous convalescence in 24 hours, or it may run as many days or even weeks. Hence it is that all manner of remedies have had a reputation in turn for the cure of rheumatism. He would not even burden the mind, he said, with an attempt to recapitulate these agents, excepting so far as related to those that had not fallen entirely into disuse. The alkalies and lemon juice were highly extolled by the English, and the former was even thought to be a specific by Dr. Fuller and some other medical authorities in the British islands.

For his part, after an extended trial, he had little faith in either, but gave the lemon juice the preference because it was sure to do no harm and was pleasant to take. The last remedy that had been recommended in the treatment of this disease, and with which he was now experimenting, was the salicylate of soda. "To this patient we will give .50 grams of this drug every hour, and see how the case will terminate." The highest temperature for the days here mentioned was as follows: Jan. 11th, 39.8; Jan. 12th, 39.2; Jan. 13th, 37; from the 14th of January to the 18th, 36.8.

As will be seen, after two days' administration of this drug the patient was in every way better; his pain had disappeared and he was entirely comfortable.

On the 14th, however, the salicylate was discontinued and .25 grams of sulphate of quinia three times a day substituted in its place.

On the 19th there was a sudden change for the worse; the left knee and elbow joints became swollen and inflamed, and the temperature showed for the next days thus: 19th, 39.6; 20th, 40; 21st, 39.8; 22d, 40; 23d, 40; 24th, 39.5. At this last date the patient was brought before the class again, and Prof. Frerichs said that in the first outset of the disease the salicylates had *seemed* to do good, but the remedy had now been given for five days, viz., from the 19th to the 24th, in doses even as high as 1 gram per hour, with no apparent benefit. He further said that if the temperature rose again to 40, and seemed inclined to remain at that point, he should begin the use of the cold bath, which he always did when drugs failed him.

For the next week this unfortunate man was used as material by Dr. Litten, to illustrate to his class in physical diagnosis, both dry and moist peri-cardial and pleuritic sounds, as well as mitral regurgitation and aortic, obstructive and regurgitant murmurs. Prof. Frerichs considers this case as an example of the failure of the salicylate treatment of rheumatism, but says, as he knows nothing better, he will give it a further trial.

I have quoted this case for the express purpose of criticising the manner in which this eminent professor used and claims to have tested the efficacy of the drug under consideration.

As the reader will see, the medicine did at first all that its advocates claim for it. At the end of two days the patient was in every way better, and seemed on the high road to recovery, but on the third day the drug was discontinued, and, as Prof. Sée would have expected, a relapse followed.

If the reader remembers my report of Prof. Sée's experiments with this agent in the disease under consideration, he will see that whenever the drug was entirely discontinued soon after an amelioration of the patient's condition, a relapse always followed. It was found that a short time after the drug was discontinued, all traces of its presence in the urine disappeared; hence the inference that it had been entirely eliminated from the system. The remedy, therefore, should not be discontinued until all danger of a relapse is past, a period not less than ten or twelve days.

Why the remedy did not produce so decided a change for the better after the patient grew worse on the 19th, can perhaps be explained by the sudden series of organic changes that occurred at this time in the heart and pleura.

W. S. CALDWELL.

BERLIN, Feb. 1st, 1879.

---

DR. A. REEVES JACKSON, the founder of the Woman's Hospital of the State of Illinois, and its surgeon-in-chief during the past eight years, has resigned his position in that institution. His example was followed by the entire surgical staff. Cause—the interference with the work of the medical board on the part of some of the board of lady managers.

The daily papers of this city have since announced that a new surgical staff has been secured for this hospital, consisting of Drs. Byford, Roler, Nelson, Merriman, Sawyer and Flood.

